# Supplementary material for: Spin Orbit Coupling in Orthogonal Charge Transfer States: (TD-)DFT of Pyrene—Dimethylaniline
Source: Molecules. 2022 Jan 28;27(3):891. doi: 10.3390/molecules27030891 (PMC8839636; doi:10.3390/molecules27030891)
Supplement: Supplementary file 1 [file molecules-27-00891-s001.zip › Supplementary-Materials-Bissesar-Williams-27-01-2022/Input files/READ-ME-INTERNAL-LAMBDA-1.docx]

This folder contains input files for ADF 2019 to determine the internal reorganization energy.

Li = Lambda Internal

CRS = Charge recombination to the singlet ground state

CRT = charge recombination to the triplet

ACN = acetonitrile solvent

NHX = n-hexane solvent

Gas = gas-phase

Acc = electron acceptor

Don = electron donor

GeoCharge = using the geometry of the charged unit

GeoNeut = using the geometry of the neutral unit

GeoTrip = using the geometry of the unit in the triplet state

GeoOpt = geometry optimization

Neutr = neutral unit, charge is zero

Charged = unit has a charge on it, (either radial cation or radical anion)

SP = single point calculation

E = energy
