# Supplementary material for: Spin Orbit Coupling in Orthogonal Charge Transfer States: (TD-)DFT of Pyrene—Dimethylaniline
Source: Molecules. 2022 Jan 28;27(3):891. doi: 10.3390/molecules27030891 (PMC8839636; doi:10.3390/molecules27030891)
Supplement: Supplementary file 1 [file molecules-27-00891-s001.zip › Supplementary-Materials-Bissesar-Williams-27-01-2022/Shivan-Davita-paper-6-12-2021-SUPPORTING-INFO.pdf]

Spin Orbit Coupling in Orthogonal Charge Transfer States: (TD-)DFT of Pyrene - Dimethylaniline

Shivan Bissesar,[a] Davita M. E. van Raamsdonk,[a] Dáire J. Gibbons,[a] René M. Williams[a]*

[a] Molecular Photonics Group, Van't Hoff Institute for Molecular Sciences (HIMS), Universiteit van Amsterdam, Science Park 904, 1098 XH Amsterdam, Netherlands.

r.m.williams@uva.nl

6 December 2021

SUPPORTING INFORMATION

Description of SI

This supporting information consists of four parts. In the first part, some extra figures are presented that contain computational output. (**appendix I**).

In the second part (**appendix II)** a detailed description of the procedures is given in which the keywords used in ADF are exemplified and highlighted.

In **Appendix III** a table of the ADF keywords is given.

In **Appendix IV** some practical aspect of the computational studies are presented.

Due to formatting issues we first repeat part of the references of the main manuscript:

.[1] .[2] .[3, 4, 5], .[6] .[7] .[8] .[9] .[10] .[11] .[12] [13] .[2,7,8,14,15] [16] ,[16,17,18,19] .[20] ,[21,22] .[23] .[24] .[25] .[26,27] .[28,29] .[30]

Appendix I:

Additional graphs

Triplet CT graphs

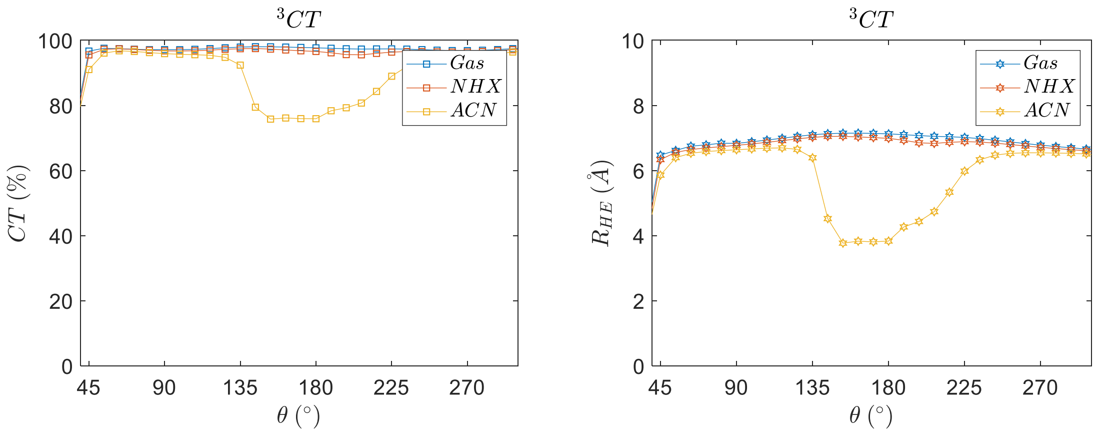

Figure S1. The conformational dependence of the charge transfer character. The descriptors are calculated as function of the dihedral angle at the optimized triplet charge transfer state (T_2) in different solvents. Note that this state does not play a role in the photo-physics of PyrDMA. The quantities are calculated with the TZP basis and the CAMY-B3LYP function. The fragment approach is used.

The electronic coupling for hole, electron transfer and charge recombination (1-2 and 2-1).

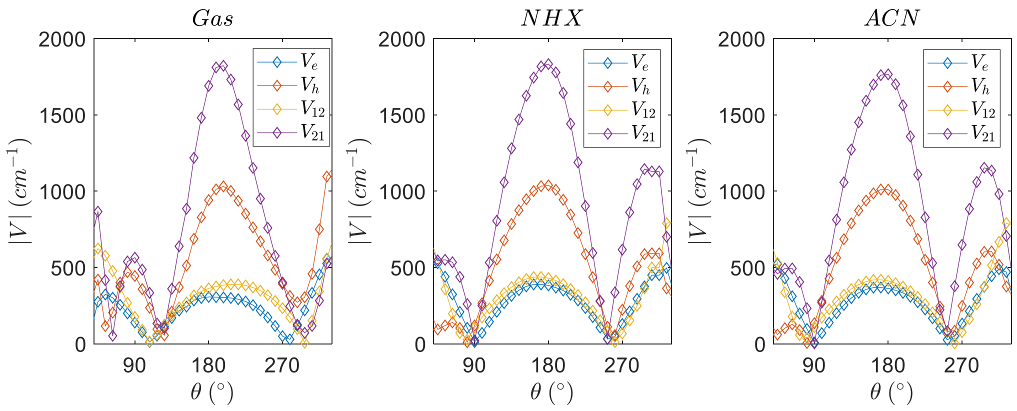

Figure S2. The electronic coupling for electron and hole transfer. Furthermore, we have the electronic coupling for charge recombination from Pyrene to DMA (1-2) and DMA to Pyrene (2-1). The couplings show similar behaviour. The quantities are computed at the S_2 geometry which was optimized with PBE/TZP. The electronic couplings are calculated with the TZP basis and the CAM-B3LYP function.

Potential energy scans are made of the optimized geometries and the sum of the total bonding energies and excitation energies were plotted as a function of dihedral angle. It has to noted that this is related to the excitation energies at the S_1 geometry. The graphs in Figure S3 show that the minimum at around 70˚ is a true minimum and not a saddle point. Furthermore, they indicate that in the energetics of the sequence of excited states that play a role in the photo-physics no strong deviations are found that indicate a large change of geometry in the excited state. It has to be noted that the energy differences between the states do not correlate to spectroscopic energies. For this, just the excitation energies are more suitable. It is also clear that around 0^0, 180^0 and 360^0 the energy of the system is so high that these conformations do not play a role in the photo-physics of PyrDMA.

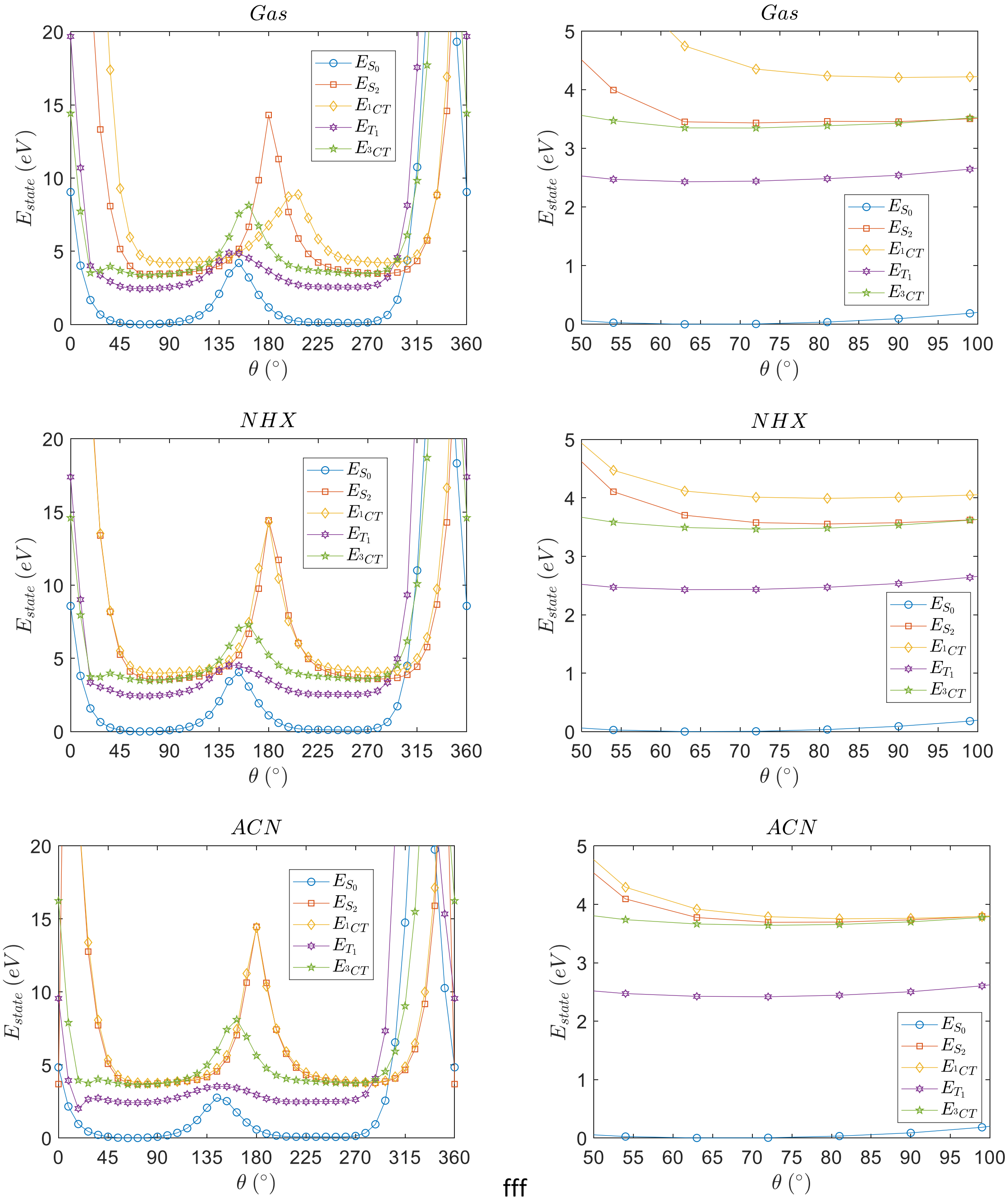

Figure S3. The total state energy (TBE + E_{exc}) curves for different geometries as a function of the dihedral angle. On the left we have the entire region from 0^0 to 360^0. On the right we zoom into the regime 50^0 to 100^0. Geometries are calculated with PBE and TZP. The quantities are calculated with the TZP basis and the CAMY-B3LYP functional.

An increase in all curves is observed at approximately 0^0 and 180^0 and 360^0 This increase is explained by a coplanar orientation of pyrene and dimethylaniline, where atoms from both fragments start approaching each other. In a more practical regime between 50^0 and 100^0, the curve changes relatively fast compared to the thermal energy. The energies of the ground state and excited states change $\sim$ 8-10 k_BT in this range, which suggests that at room temperature the conformations are constrained to one geometry.

Appendix II

In this section, we describe the computational steps with their detailed background and keywords in ADF.

Geometry Optimizations

The first step in the study of the SOCT-ISC mechanism is to perform geometry optimizations. Geometry optimizations need to be performed for the ground-state (GS or S_0), singlet (S_n), and triplet (T_n) excited states. Both DFT, TD-DFT, and DFTB calculations are required for the calculations. First, the geometries that can be calculated with DFT will be discussed as well as the keywords that are of importance. Secondly, the TD-DFT calculations will be discussed and lastly, the TDFT calculations that are needed for the Huang-Rhys factors are discussed.

DFT calculations can not only be performed for the S_0, but also for the T_1-state as it is the lowest triplet manifold and can therefore be optimized as if it was a normal ground-state but with triplet spin multiplicity.[31] Other than the multiplicity, the input will be the same. General keywords will not be discussed, but there are some keywords more specific for these calculations.[32] Solvation effects should be included with the keyword *COSMO*. Since calculations on the SOC will be performed, the relativistic scalar *ZORA* needs to be included. These geometry optimizations are performed in the *unrestricted* mode which allows changing the *SpinPolarization* to 2.0 (triplet multiplicity). This calculation should be combined with the *noncollinear* approximation, in which the spin-polarization can have a different direction in every point of space. In a noncollinear approximation, the symmetry should be changed to *NOSYM*. PES point characterizations (*PESPointCharacter Yes*) can be included in the calculation to validate the geometry, but should be turned off in later calculations.[33]

For TD-DFT calculations, more specific keywords need to be included. Thus, geometry optimizations can be performed of higher S_n and T_n states. First, the type of excitations should be selected: for singlet-singlet excitations, this corresponds to the *ONLYSING* keyword.[32] To perform an excited state geometry optimization, the *EXCITEDGO* block should be included. Within this block, the excitation for which the gradient is to be evaluated, should be included: both the symmetry label and the n-th state of interest. Also, *Singlet* should be included if a singlet-singlet excitation is desired, and *Triplet* if a singlet-triplet excitation is of interest.

As (TD)-DFT calculations take up a lot of computer power, a faster and cheaper method can sometimes be a good alternative, although caution should be taken. DFTB calculations look very similar to DFT calculations except the engine should be changed from ADF to DFTB. Furthermore, a model and parameter directory should be selected based on literature aligning with the system of interest. More information on when DFTB is used, and with which keywords, can be found in the section about CR_T.

Initial Charge Separation

In this part, different parameters that can be calculated with ADF will be discussed to give more insight into the mechanism and in particular into CS that needs to be optimal for efficient SOCT-ISC.

There are different theoretical approaches for studying intramolecular electron transfer of which the Marcus theory gets the most attention.[34] In his theories, Marcus distinguishes two types of reactions: adiabatic and diabetic electron transfer processes. The energetic plots in diabatic electron transfer can be described as shown in equation S1.[22]

$$k_{ET} = \frac{2\pi}{\hbar}\frac{1}{\sqrt{4\pi\lambda k_B T}}|V_{CT}|^2\, exp\left(-\frac{(\Delta G+\lambda)^2}{4\lambda k_B T}\right) \qquad (S1)$$

$\hbar$ and k_B represent the Planck and Boltzmann constants, respectively. ΔG is the Gibbs free energy change between reactants and products in equilibrium. The energy that is needed to distort the product state to adapt to the geometry of the reactant state is represented by the reorganization energy, λ. The magnitude of V_{CT} indicates the coupling strength between the initial and final state and is called the electronic coupling matrix element.

The electronic coupling is the same as the effective or generalized charge transfer integral (J_{eff}).[35] The charge transfer integral corresponding to the electronic coupling between the local excited (LE) state and the CT-state can be calculated with the quantum chemical program ADF.[35,36,37] For calculating the charge transfer integrals, a fragment approach is used. The fragment approach makes use of MOs on the individual fragmental molecules as a basis set of the calculations on a system containing two or more fragmental molecules. For example, the donor and acceptor of an organic dyad (LE state geometry) are split and form two fragments. The two fragments are first computed separately, followed by a computation that calculates the interaction between the two fragments. The *TRANSFERINTEGRAL* keyword should naturally be included, otherwise, it will not be determined. The charge transfer integrals, site energies, and overlap integrals between the two fragment orbitals are calculated. The charge transfer integrals can simply be found in the output. The integrals are by default only calculated for the HOMO and LUMO of the fragments. However if needed, the matrix element and overlap integrals of all fragment orbitals can be included with the print statement *FMATSFO*.[32,15]

Another parameter from the Marcus theory that can be calculated is the reorganization energy (λ). The reorganization energy consists of an external (λ_{ext}) and internal (λ_{int}) component.[38] The λ_{ext} can be studied in the classical way as proposed by Marcus.[39] The focus is on calculating λ_{int}, which measures the change in energy that is needed to rearrange the nuclei of the dyad upon charge separation from a neutral to a charged state. The reorganization energy for the electron donor (λ_D)

and the electron acceptor (λ_A) of the molecules can be calculated, in a quantum chemical fashion, based on the four-point method developed by Nelsen, following equation S2, S3 and S4.[38,40]

$$\lambda_{int} = \lambda_D + \lambda_A \tag{S2}$$

$$\lambda_A = \frac{1}{2}[(E_0^- - E_-^-) + (E_-^0 - E_0^0)] \tag{S3}$$

$$\lambda_D = \frac{1}{2}[(E_0^+ - E_+^+) + (E_+^0 - E_0^0)] \tag{S4}$$

For charge separation this can be clarified by (superscript is charge plus unpaired electron):

$$\lambda_A = \frac{1}{2}[\left(E^{-\cdot}{}_{\substack{gs \\ coord}} - E^{-\cdot}_{\substack{anion \\ coord}}\right) + \left(E^0_{\substack{anion \\ coord}} - E^0{}_{\substack{gs \\ coord}}\right)]$$

$$\lambda_D = \frac{1}{2}\left[\left(E^{+\cdot}{}_{\substack{gs \\ coord}} - E^{+\cdot}_{\substack{cation \\ coord}}\right) + \left(E^0_{\substack{cation \\ coord}} - E^0{}_{\substack{gs \\ coord}}\right)\right]$$

The E_0^+ (E_0^-) represents the energy of the cation (anion) at the optimized geometry of the neutral molecule.[53] In the same manner, the other terms can be explained: E_+^+ (E_-^-) represents the energy of the cation (anion) calculated at the optimized geometry of the cation (anion); E_+^0 (E_-^0) represents the energy of the neutral molecule calculated at the optimized geometry of the cationic (anionic) state; E_0^0 represents the energy of the neutral molecules at the optimized geometry of the ground state.

The described energy states are, for clarity, shown in Figure S4. The neutral state of the cation and anion is the locally excited state S_2, which is the excited state S_1 of pyrene.

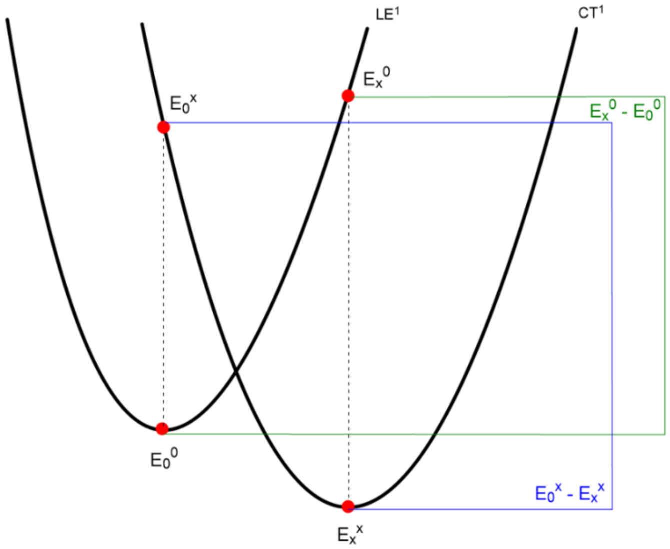

Figure S4. PES of the LE and ¹CT states. With x being + or -.

As just explained, the reorganization energy can be determined by calculating all the different energies with the use of TD-DFT. For this, four geometry optimizations need to be performed for the neutral fragments and the anionic, and cationic fragments. Thereafter, four single point calculations need to be done. For example, take the geometry of the cationic donor fragment and perform the single point calculation as if it was the neutral donor fragment. In ADF, this can be done by taking the optimized geometry of the cationic fragment and changing the charge from 1 to 0 and setting the spin polarization to 0 as well.[32] To change the spin polarization, the calculations should be unrestricted. This can be repeated to gain the needed energies on the PES, which correspond to the energies of equations S3 and S4.

The last parameter that is needed for the Marcus equation is the Gibbs energy (ΔG). The Gibbs energy can be regarded as the energy difference between the minima of the excited state potential energy surfaces (PESs).[37,38] The entropic and zero-point energy contributions are neglected as it is assumed that their contributions are small compared to the energy difference (ΔE). The Gibbs energy can be calculated by taking the difference in energy of the two states of interest. The excitation energies can be taken to calculate the Gibbs energy as the excitation energies can be optimized with a functional that can better describe charge transfer states (see Considerations & Limitations).[37] The excitation energies can be calculated very similarly to the geometry optimizations, but the *EXCITEDGO* block is not included.

In the output, charge transfer descriptors are provided of two methods. One of the methods is the charge-transfer diagnostic overlap LAMBDA (Λ) developed by Peach, Tozer *et al*.[41] They studied the correlation between the excitation energy error and the spatial overlap between the occupied and virtual orbitals that are involved. Small Λ and large errors can be associated with each other and vice versa, however, this is dependent on the functional used and vanishes with hybrid and range-separated hybrids. The vanishing of this relation can be used to determine the reliability of a certain excitation energy. Λ is a value between 0 and 1, where a small value corresponds with a long-range excitation and a large value with a short-range excitation. A high value for Λ means a high overlap, which indicates that in the excitation the occupied and virtual orbitals can be expected in similar parts of space. Local excitations with small CS can be assigned to these high Λ values. Also visible in the output is R_{HE} or Δr, the average distance between the electron-hole separation upon electronic excitation and is related to the nature of the excitation: a large distance can be found for CT excitations and a short distance for valence excitations. The other method for charge transfer descriptors is based on that of Plasser, Lischka, *et al.*[23] The charge transfer descriptor CT_{AT} gives insight into the charge-transfer character. The final and initial orbitals should be on different fragments. The value for the CT

character has a value between 0 and 1, where 0 is an excited state which can be seen as a locally excited state with no CS, and 1 of a state which is totally charge separated.

Charge Recombination to The Ground-state

After CS, CR can occur and the most obvious one is the spin-allowed CR from the charge transfer state to the ground state. CR_S or CR_T are largely dependent on each other because the decay of one influences the photophysical pathway of the other.[13] When CR_S is efficient, CR_T will not be efficient and vice versa. It is for this essential that CR_S is suppressed to enhance the efficiency of the SOCT-ISC. Suppressing the CR_S pathway is possible in the inverted region of the Marcus theory.

The Marcus theory describes three regions for electron transfer of which one is the optimal region which states that barrier-less electron transfer can occur if $\lambda = -\Delta G$.[42] The situation before the optimal is reached is the normal region, where the rate of electron transfer increases with an increasing driving force(here $\lambda > -\Delta G$). The inverted region can be counterintuitive, but in this region, the rate of electron transfer decreases as the driving force further increases ($\lambda < -\Delta G$).

The thermodynamics of CR_S from a CT state often fall in the inverted region.[43] If the energy gap between the CT and S_0 state is large, the driving force will increase.[7] The driving force is significantly increasing in a way that the rate of CR_S is decreasing in the inverted regime. As a result, the CT state is longer lived. To suppress the CR_S, the energy gap should be tuned. One way of tuning the energy gap is via the solvent.[44] Charge separated states are polarized and can be stabilized by polar solvents, a more stabilized molecule is lower in energy and sequentially the energy gap between the S_0 and CT state is smaller. The energy difference can again be taken from the excitation energies for a better description.

The inverted region begins when the reorganization energy becomes smaller than the driving force. In the last paragraph, the focus was on the driving force, but the reorganization energy can also be tuned.[34] Certain acceptors have already relatively small reorganization energies and the inverted region will appear earlier in the kinetic energy curve for these acceptors. This results in fast CS and a slower CR_S.[45] An example of an acceptor with a small reorganization energy is fullerene. However, selecting a structure based on its reorganization energy may not always be ideal. Studying the reorganization energy is of course best done if it is possible to measure its value. The reorganization energy can be calculated via the same method as discussed in the section Initial Charge Separation using equations S2, S3 and S4. The neutral state of the cation and anion is the ground state of the cation and anion. Studying an interaction between two states is often studied from the precursor state. This means that it is not possible to take the results from the previous section, where a LE state was

used, while here a CT-state is needed to study the CR_S process. A new calculation is necessary for which only the coordinates have to be changed.

This process will ideally take place in the inverted region, which asks for a more sophisticated description of the Marcus theory and its reorganization energy (see later). To determine the rate of CR_S, equation S1 can be used. The electronic coupling for charge separation can be calculated via the fragment approach.[37] The output of this calculation does not only give the electronic coupling for electron transfer, but it also gives the electronic coupling for charge recombination processes that represent the probability.[22,32] If CR_S can be suppressed, CR_T can become the dominant pathway.

Charge Recombination to The Triplet-state

Transitions between singlet and triplet states are forbidden in a non-relativistic framework, however, intersystem crossing from a singlet to a triplet state is possible in the presence of spin-orbit coupling.[46] Spin-orbit couplings in heavy-atom free dyads are very small compared to structures that include heavy atoms. For this reason, these kinds of systems can be treated as a perturbation of the system's electronic structure. It is challenging to calculate the SOC factor in both an effective and accurate way.[1]

Using quantum chemistry, it is possible to gain more insight into SOC as it is the result of the spin-operator expressed by its Hamiltonian.[2,47] This spin-orbital Hamiltonian describes the interaction between the spin and orbital motions of an electron and induces singlet and triplet excitations. The coupling of the spin and orbital momenta of the nucleus and electron is described by the Hamiltonian (equation S5).

$$\hat{H}_{SO} = \alpha_{fs}^2 \sum_{\mu}^{N} \sum_{m}^{n} \frac{z_\mu}{r_{i\mu}^3} \overrightarrow{L_m S_m} \quad \text{(S5)}$$

α_{fs} is the fine structure constant, the effective nuclear charge for nucleus μ is represented by Z_μ. L is the orbital momentum and S the spin momentum. The distance between the nucleus and electrons is represented by r.

The most outstanding feature of the Marcus equation is the inverted region effect, in which the electron transfer kinetics will slow down because of $\Delta G > \lambda$. However, in the inverted region, the rates are higher than expected if the classical equation is used.[42] This deviation can be explained by quantum chemical vibrational effects that start to play a role. The PESs of two states at the minima can be described as vibrational normal modes that lead to vibrational eigenfunctions within a quantum mechanical description. An alteration of the classical Marcus equation is proposed; following Fermi's Golden Rule, the Franck Condon Weighted Density of states (FCWD) can be introduced.

Fermi's golden-rule for radiation-less transitions can be used to describe the intersystem crossing rate, k_{ISC} with $\langle {}^1\Psi_i{}^0|H_{SO}|{}^3\Psi_F{}^0\rangle^2$ being the SOC matrix element between the initial and final state of interest (equation S6). The FCWD after being evaluated in the framework of Marcus-Levich-Jortner theory can be expressed as visualized in equation S7.[48]

$$k_{ISC}^{IF} = \left(\frac{2\pi}{\hbar}\right)\langle {}^1\Psi_1{}^0|H_{SO}|{}^3\Psi_F{}^0\rangle^2\,[FCWD] \quad \textit{(S6)}$$

$$FCWD = \frac{1}{\sqrt{4\pi\lambda k_B T}}\sum_{n=0}^{\infty} e^{-S}\frac{S^n}{n!}exp\left[-\frac{(\Delta E+n\hbar\omega+\lambda)^2}{4\lambda k_B T}\right] \quad (S7)$$

This extension of the semi-classical Marcus theory considers two limiting cases.[49] The first, is the consideration of low-frequency vibrations which leads to results similar to the outcomes from the previously discussed classical Marcus theory that can be described in terms of the reorganization energy as defined in the classical Marcus theory. The second consideration, is that of the high-frequency modes and nuclear tunneling effects. These modes can be summarized in a single effective mode with ω as frequency, which is visualized in equation S7. The classical equation can be recognized in the semi-classical one, but there is a new factor; S, which is the Huang-Rhys factor of this effective mode. It is a measure of the strength of electron-phonon coupling. Upon photoexcitation, the equilibrium positions of the nuclei are displaced, the Huang-Rhys factor is associated with this phenomenon. The individual high frequency normal modes have corresponding frequencies ω_i and Huang-Rhys factors, S_i, of which the effective Huang-Rhys factor can be calculated (see equation S8 and S9).

$$S_{eff} = \sum_i S_i \quad (S8)$$

$$\omega_{eff} = \sum_i \frac{\omega_i S_i}{S_{eff}} \quad (S9)$$

DFTB and FCF methods implemented in the ADF package can be used to calculate the Huang-Rhys factor.[50] DFTB geometry optimizations of the two excited states need to be performed. The keyword *TD-DFTB gradient* should be included as it orders the program to calculate analytical gradients for the TD-DFTB excitation energies and the keyword, *SingleOrbTrans,* will calculate single orbital transitions.[51] This allows for optimization of the excited state geometries and makes it possible to calculate the Frank-Condon Factors with the FCF program, which computes the effective S_{eff} and ω_{eff}. ω_{eff} is

often assumed around a value of 1500 cm^{-1}, however, DFT calculations show that the value for ω_{eff} can easily be around 800-900 cm^{-1}, significantly lower.[22] The value for the Huang-Rhys factor for ω_i can mostly be found between 0 and 1. Assuming ω_{eff} can therefore have a large consequence on this value. Using DFT calculated values for molecular systems can give a better approximation.

As explained before, the reorganization energy consists of an internal and external component, and the focus is again on the internal component. The internal reorganization energy for the transition from CT^1 to T_1 can be defined similarly as visualized in equations S10, S11 and S12.[38,52]

$$\lambda_{int} = \lambda_D + \lambda_A \tag{S10}$$

$$\lambda_A = \frac{1}{2}[(E_-^0 - E_0^0) + (E_0^- - E_-^-)] \tag{S11}$$

$$\lambda_D = \frac{1}{2}[(E_+^0 - E_0^0) + (E_0^+ - E_+^+)] \tag{S12}$$

For triplet formation by charge recombination, where the triplet localizes on the acceptor, this can be clarified by (superscript is charge plus unpaired electron):

$$\lambda_A = \frac{1}{2}[\left(E^0_{\substack{anion \\ coord}} - E^0_{\substack{triplet \\ coord}}\right) + \left(E^{-\cdot}_{\substack{triplet \\ coord}} - E^{-\cdot}_{\substack{anion \\ coord}}\right)] \tag{13}$$

$$\lambda_D = \frac{1}{2}[\left(E^0_{\substack{cation \\ coord}} - E^0{}_{\substack{gs \\ coord}}\right) + \left(E^{+\cdot}{}_{\substack{gs \\ coord}} - E^{+\cdot}_{\substack{cation \\ coord}}\right)] \tag{14}$$

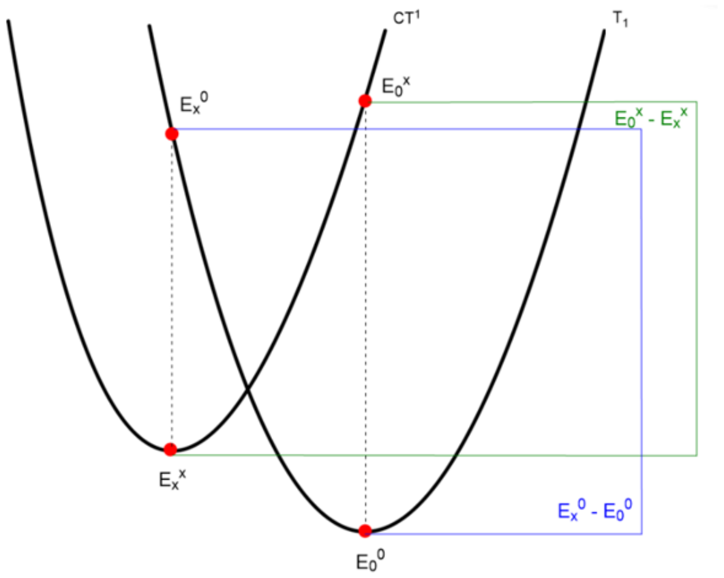

Figure S5. PES of the CT^1 and T_1 states. With x being + or -.

The coordinates (subscript) belong to the radical anion, the radical cation or the triplet. For the radical anion, the charge has to change to -1, and the spin-polarization to 1 (one unpaired electron, formally a doublet state). For the radical cation, the approach is similar. The definitions of the equations stay the same, but the donor and acceptor should be studied differently because triplet formation occurs either on the donor or acceptor.[2] For the fragment on which the triplet is formed, the energies as shown in Figure S5 can be used in combination with the definitions that are discussed above. For the other fragment, the CT state and S_0 should be considered, just as for CR_S. The E_0^+ (E_0^-) represents the energy of the cation (anion) at the optimized geometry of the neutral molecule.[53] In the same manner, the other terms can be explained: E_+^+ (E_-^-) represents the energy of the cation (anion) calculated at the optimized geometry of the cation (anion). E_+^0 (E_-^0) represents the energy of the neutral molecule calculated at the optimized geometry of the cationic (anionic) state. E_0^0 represents the energy of the neutral molecules at the optimized geometry of the ground state. Alternatively to this method, is the method performed by Samanta *et al.*,[49] where the reorganization energy during reversed-ISC can be defined as the energy difference between E_0^x and E_x^x.[49] This alternative can be studied next to the proposed method, but considering E_0^0 and E_x^0 in ISC from CT^1 to T_1.

λ_{int} consists of low- and high-frequency modes and can be represented as a sum of the contributions of the individual vibrational normal modes:

$$\lambda_{int} = \sum\lambda_i = \sum\hbar\omega_i S_i \tag{15}$$

$$\lambda_i = \frac{k_i}{2}\Delta Q_i^2 \tag{16}$$

The normal mode is represented as i, k_i is the force constant and ΔQ_i is the coordinate displacement along the normal mode. Using the outcomes of the DFTB and FCF calculations will help to determine the reorganization energy for CR_T. This is another method that can be used to calculate the reorganization energy. By defining an effective frequency and Huang-Rhys factor, it is possible to consider one effective degree of freedom and it is often not necessary to consider more modes.[52] The Huang-Rhys factor and the reorganization energy seem to change only a little compared to the energy gap and SOCME, but can still give useful insight from a fundamental perspective.

The energy gap between the two states is the parameter that changes most significantly, and together with the SOCME, it determines the ISC rate.[48] Following El-Sayed's rules, the rate for ISC in radiation-less transitions is larger when a change of MO type is present. Thus, an increase in the spin-orbit coupling between the S_n and T_n can lead to efficient ISC.[9,33] Fermi's golden rule indicates that a larger energy gap between two states reduces the coupling matrix elements of the two states which will result in slower kinetics and ISC.[54]

In the papers of Zhu and colleagues, and Samanta *et al.*,[49] the SOC matrix element was computed by considering the three degenerate T_1 triplet states ($m = 0, \pm 1$) as is shown in equation S17.[31,49]

$$V_{SOC} = \langle S_1|\hat{H}_{SO}|T_1\rangle = \sqrt{\Sigma_{m=0,\pm1} \langle S_1|\hat{H}_{SO}|T_1^m\rangle^2} \qquad \text{(S17)}$$

Calculating the SOC between the ^{1}CT and T_1 state can be done using TD-DFT calculations and for more accurate results, solvent effects should be considered. They studied the SOC operator within the zeroth-order regular approximation (ZORA) visualized in equation S18, where c represents the speed of light, V the Kohn-Sham potential, p the linear momentum operator, and σ the Pauli spin matrix vector.

$$\hat{H}_{SO} = \frac{c^2}{(2c^2-V)^2}\sigma(\nabla V \times p) \qquad \text{(S18)}$$

First, the excited state geometries should be calculated (see also first section). For the SOC calculation in ADF, a few keywords are worthy to mention (see also appendix III). The type of excitation should be spin-orbit (perturbative) or simply the *SOPERT* keyword should be included, which includes singlet-singlet and singlet-triplet excitations and includes spin-orbit perturbatively. Also, *NTO*s and *charge transfer descriptors* should be included.[49] As explained, the triplet has three sublevels, thus three complex values can be expected between the CT state and the three sublevels. However, to make it easier the output file contains a summary of SOCMEs as the root-mean-square average. To include the SOCME in the output file, the keyword *PRINT SOMATRIX* should be included in the input file. Tamm-Dancoff approximation (*TDA*) should be included in the input file to calculate excitation energies, which can be used to determine the energy gap between the ^{1}CT and T_1 state.[1]

Discussing quantum chemical equations makes this topic hard to follow and to make it chemically more understandable, the SOC transition can be seen as a linear combination of the scalar singlet-singlet and singlet-triplet excitations as described by Ronca and colleagues.[55] From this perspective, the oscillator strength of the SOC (f_{SOC}) can be expressed as a singlet-triplet transition that borrows intensity from the coupled singlet – singlet state, as shown in equation S19.

$$f_{SOC} = \frac{\langle \Psi_S|\hat{H}_{SO}|\Psi_T\rangle}{E_T - E_S} f_s \qquad \text{(S19)}$$

The f_{SOC} is directly correlated to the strength of the coupling, the SOC matrix element, and is inversely correlated to the energy differences between the coupled triplet and singlet states. It is often mentioned in the overviews of parameters of the excitations in studies focusing on the SOCT-ISC mechanism.[56]

Computational calculations can predict the energy levels of the lowest triplet states. Even though these triplet states can be energetically accessible, CR to these energy levels is in principle forbidden, because of spin conservation.[54] Research done by Letrun and colleagues found triplet states that lay below the S_1 are localized on either the donor or acceptor of the dyad.[57] If CR occurs between a donor and an acceptor with their HOMO and LUMO localized on other parts of the dyad, the electron will undergo a change. When the donor and acceptor are in an orthogonal orientation to each other, there will also be a change in angular orbital momentum, which will induce a large SOC, allowing CR_T.

The orthogonality often meant is the orthogonality between the two molecular planes, as just discussed, but there is also research that mentions an extra orthogonality of the nodal planes that may affect the ISC positively.[2,10] This is in agreement with the idea that the $\widehat{\mathrm{H}}_{\mathrm{SO}}$ operates between the initial and final states and reaches a maximum when the orbitals are orthogonal.

Thus, studying the HOMO (-) and LUMO (+) MOs can give insight into whether or not a large SOC can be expected for the system. This can also be used for proper molecular design. Multiple MOs may contribute equally to an excited state, these MOs can be transformed to a Natural Transition Orbital (NTO).[39,51] With the NTO, the qualitative description of the electronic transition can be simplified and it gives insight into the localization of excitations. The geometry that is used for the SOC calculations should also be the geometry at which the NTOs are compared. Thus, if the SOCME is calculated from the optimized CT state geometry, the NTOs of the CT and T_1 state should be compared at the geometry of the CT state.[49]

Triplets decay to the Ground-state

After charge separation and charge recombination to the triplet state, the triplet state is populated, but how long will it take before it decays back to the ground-state? The first step in calculating the radiative decay from T_1 to S_0 is to optimize the geometry of the T_1-state. The next step is to perform a SOC-TD-DFT calculation.[32] This can be done similarly as was done for the ISC to the triplet state, thus via a perturbative SOC calculation. Both the oscillator strengths and radiative lifetimes will be printed in the output. The radiative lifetime and oscillator strength are related according to the following equation.

$$\frac{1}{\tau} = \frac{2(\Delta E)^2 f}{c^3} \tag{20}$$

The radiative lifetime corresponds with τ, the oscillator strength with f, ΔE is the excitation energy and c is the speed of light. The atomic unit is used in this equation. The input file differs slightly from the perturbative SOC calculation performed before: *TDA*, *NTO,* and *DESCRIPTORS* should be left out of the input file. The excitation block and the *SOPERT* key should be included. The *PRINT SOMATRIX* keyword can be included to print the whole SOCME. The SOCME is an important parameter as it influences the oscillator strength and therefore, the radiative lifetime. SOC was important for the normally forbidden transition to the triplet state, but is again important as decay from a triplet-state to the ground-state is once more a spin forbidden process, also known as phosphorescence.[58] In the introduction, the heavy atom effect was explained, and with an increase in atomic number the SOC will increase.[59] Studies of porphyrin derivatives substantiate this phenomenon and show high rates of phosphorescence.[60] In the same research, they also have shown that heavy-atom free porphyrin derivatives can emit very weak phosphorescence, even though they have efficient ISC to the triplet state. This combination of efficient ISC and slow decay and, therefore a long-lived triplet state production can be very useful in a variety of applications, such as photodynamic therapy. The lifetime of triplets are calculated and presented in Table S1.

Table S1. The lifetime of the triplet state of PyrDMA in the different environments. The geometries are calculated with the TZ2P basis and CAMY-B3LYP functional.

Solvent	τ (s)
Gas	11.79
NHX	1.41
ACN	20.26

Triplet yield

We neglect the intersystem crossing rate to the ground state. This gives the following equation for the triplet yield.

$$\phi_T = \frac{\frac{k_{CS}k_{CRT}}{k_{CS}+k_{CRT}}}{\frac{k_{CS}k_{CRT}}{k_{CS}+k_{CRT}}+\frac{k_{CS}k_{CRS}}{k_{CS}+k_{CRS}}} \quad (21)$$

$$\phi_T = \frac{k_{CRT}(k_{CS}+k_{CRS})}{2k_{CRT}k_{CRS}+k_{CS}(k_{CRS}+k_{CRT})} \quad (22)$$

The equation for the triplet yield suggests that this yield is efficient for high rates of charge separation and charge recombination to the triplet. The rate of charge recombination to the ground state has to be low.

Solvent effects

Polar solvents are better at stabilizing charged molecules, making charge separation more favorable in polar solvents. However, a stabilized CT energy level means it is lower in energy, which stimulates CR to the ground state.[44] In less polar solvents the CT energy level increases, enlarging the energy gap between the CT and S_0 state, and thus decreasing the CR to the ground state.[7] The solvent plays an important role and should be considered in computations. The ADF program makes use of the conductor-like screening model (COSMO) to describe any solvent effects.[61] As parts of the calculations are TD-DFT calculations, it should be mentioned that the induced electronics will affect the COSMO surface charges. However, geometry optimizations of the excited states should work fine, because the solvent dielectric has time to respond.

Considerations & Limitations of ADF

The functional, basis set and other technical settings may affect the accuracy of the calculation. Furthermore, research has shown that the transfer integrals depend greatly on the functional that is used.[62] This is also the case for the site energies and reorganization energies. Different functionals may be more accurate for the different components. Both DFT and TD-DFT calculations need to be performed. TD-DFT results are often altered by the self-interaction error.[63] This error can have significant alterations on the energies and electronic structure of the excited states. Hybrid methods can be selected to reduce the self-interaction error, but the same parameters are sensitive to the amount of exact exchange that is incorporated in these hybrid methods. An alternative is the range-separated hybrid DFT method where the amount of exact exchange increases with the electron-electron distance, which restores the long-range asymptotic behavior of the potential. For this reason, range-separated hybrids are more accurate in describing electronic excitations. This is especially important for describing CT states, therefore, calculations for the excitation energies are often performed with TD-DFT with the range-separated CAM-B3LYP functional.[13,37] Range-separated hybrids have three parameters included that can be further be optimized for a certain system.[64] This may affect the accuracy of the calculations and can therefore give an even better approximation of the parameters studied in the past sections. It should be noted that CAM-B3LYP cannot be used to perform excited state geometry optimizations, but CAMY-B3LYP can. However, this functional is far less often used in similar studies but can be considered to be used in a benchmark to study their accuracy.[38] Hybrid functionals are often used in computational studies on the CT state as well as on the SOCT-ISC mechanism. Hybrid functionals are often preferred over a range-separated functional perhaps because of the CPU time, which is already increased for hybrid functionals, but even more for range-separated functionals. To reduce CPU time, the integration can be set to a lower accuracy e.g., Becke Normal. Frequently, the B3LYP functional is used in combination with the TZP basis set.[36,65] In the research of Monti *et al.*, they performed a benchmark with different hybrid functionals and B3LYP fitted the experimental results best and it was also used in combination with the TZP basisset.[37] Others also found the hybrid functional PBE0 to fit well.[33] It is important to perform benchmarks to make sure that the functional and basis set are accurate and it is recommended to check with literature on specific structures. As hybrids and range-separated functionals are proposed, it should be mentioned that NTOs will only be calculated if TDA is included. Also, the ZORA two-component Hamiltonian should be included in order to evaluate the SOCME.[49,66] Comparing experimental results with computational findings can also help substantiate the accuracy of the proposed method.[67]

This section has shown the possibilities of computational tools implemented in ADF for the study of the SOCT-ISC mechanism. Three fundamental pathways are of essence in this mechanism and each of these has been discussed. A general overview of the CS, CR_S, and CR_T pathways was briefly highlighted and was thus followed by an overview of the computations that could be done on the different components in the specific pathway. Throughout the text, the Marcus theory, in particular the inverted region, was named and discussed and the importance of this theory should not be neglected. It forms the basis of this article together with the rules of El-Sayed. During initial CS, the electronic coupling can be seen as the most important factor that influences the rate of separation and ADF gives a unique feature of studying this parameter via a fragment approach. The electronic coupling is also of essence during CR_S, but also the Gibbs energy and reorganization energy play a significant role in shifting the process in the inverted region. The SOCME that can be calculated with the same chemical software is the parameter that determines the rate of intersystem crossing for the largest part. The triplet decay gives more insight into the lifetime of the triplet state. Throughout the text, the importance of any solvent effects has been clarified and a section was dedicated to the consideration of solvent effects in DFT calculations, focusing on the COSMO method. Other considerations and limitations were also discussed. An overview of the possibilities with ADF was made and a method to study the SOCT-ISC mechanism is given. Following this method can give new insight in the complex SOCT-ISC mechanism as it includes different aspects that can play a role in the mechanism. Future computational studies should emphasize and substantiate the relevance of including all aspects while following the proposed method. The best course of action is to compare the results with experimental findings and optimize the method where needed.

Appendix III: ADF Keywords

This table contains ADF Keywords that are of importance for the study of the SOCT-ISC mechanism. Each subsection contains the keywords that are special for that section (keywords will not be repeated if required to be included in different sections).

Geometry Optimization	
General + S_0 (DFT)	
noncollinear	In this approximation, the spin-polarization can have a different direction in every point in space.
Unrestricted	α & β-spin may be spatially different
RELATIVISTIC Scalar ZORA	Includes the relativistic scalar ZORA
SOLVATION	Includes solvent effects (COSMO)
SYMMETRY NOSYM	No symmetry (forced)
T_1-specific (DFT)	
SpinPolarization	Set to 2.0 to change spin multiplicity (triplet)
PESPointCharacter	Checks if PES point is a saddle point or minimum
S_n & T_n-specific (TD-DFT)	
EXCITEDGO	Excited state geometry optimization
ONLYSING	Only singlet-singlet excitations
ONLYTRIP	Only singlet-triplet excitations
Charge Separation	
Print FMATSFO	Matrix element & overlap of all fragment orbitals
TRANSFERINTEGRALS	Includes charge transport properties
CHARGE n	n = charge of molecule/fragment
SPIN POLARIZATION n	Spin-α electrons in excess of spin β
CR to the triplet state	
TDA yes	Includes the Tamm-Dancoff approximation
NTO	Includes Natural Transition Orbitals
SOPERT	Type of excitation: Spin-Orbit Perturbative
DESCRIPTORS	Includes Charge Transfer descriptors
Print SOMATRIX	Shows SOCME in output
(TD)-DFTB	
SingleOrbTrans	Single orbital transitions will be calculated
TDDFTBGradient	Allows optimization of excited states. Needed for FCF to run

Appendix IV

Practical Experimental aspects

Starting coordinates
The starting coordinates for PyrDMA were made with geometry optimisations and building in Spartan, DFT minimized C24H19N, RB3LYP, Basis set 6-31G(D), E(bond) = -980.786369, volume (CPK): 353.75 Å^3, E(HOMO) = -5.31 eV, E(LUMO) = -1.55 eV. The file was saved as *Start-Coord-Pyr-DMA.mol2*.
In case the procedure has to repeated for a different molecule, type the name of the molecule in the search bar in the ADF GUI. If the molecule is not available, the molecule can be built with the carbon, oxygen, hydrogen and nitrogen tools. Bond order can be changed by clicking on the bond and selecting the "bond order". Once the molecule is built click on the geometry optimisation tool on the bottom right bar in the ADF GUI. The starting coordinates are now obtained. The starting coordinates can then be exported as a .mol2 by going to "file" and then "export coordinates". If possible it is good to use an CIF file or coordinate file from a crystal structure as a starting point.

Geometry optimisations

5 geometry optimisations need to be performed in 3 different solvents. To calculate the geometries, go to the ADF GUI. Go to "File" and then "Import Coordinates". Set "Task" to "Geometry Optimization" and choose the desired basis under "Basis set" and exchange correlation under "XC-Functional". Next, select "Relativity" and choose the "Scalar" option. Set "Frozen core" to "None" and "Numerical quality" to "Good". Click the "Model" panel and select "Solvation". Next choose the solvation method, which is "COSMO". Select the solvent from "COSMO solvent". Save the file with a proper name corresponding to the molecule, geometry and solvent. Press *Ctrl + R* to run the geometry optimisation.

For excited state geometry optimisations, repeat the same steps as above and go to "Properties" and then click "Excitations (UV/Vis), CD". For different types of excitations, select "SingletOnly" for the singlet excited state and "TripletOnly" for the triplet excited states. Select "TDA" and then "Yes". Set the "Number of excitations" to 5. Go to calculate and tick "NTOs" and "Charge transfer descriptors". Next go to "Properties" and select "Excited State Geometry". Enter the state and symmetry under "Excitation". For S_1, this would be *1A.* Set the spin type to "Singlet" or "Triplet" depending on the excited state. At the end of the calculation, the coordinates of the geometries can be extracted from the logfile in the final geometry cycle. Copy and Paste these coordinates in the ADF GUI and export as a .mol2 file with a proper name corresponding to the molecule, geometry and solvent.

Geometry optimisations have been done with two different methods.

- Method 1: Geometries calculated with TZP basis and PBE functional. Triplet geometries are calculated by setting the charge and spin polarisation to 0 2. TDA is neglected for excited state geometry optimisations.
- Method 2: Geometries with TZ2P basis and CAMY-B3LYP functional. All excited state geometries are calculated with EXCITEDGO.

The geometries optimisations generate .run files which can be modified as an input file for calculating the geometry optimisations on the Lisa server. If the calculations need to be done on the Lisa server, simply use the standard input files *PyrDMA-Geometry-S0.txt*, *PyrDMA-Geometry-S1.txt*, etc. Change the starting coordinates to the coordinates of the new molecule by Copy and Pasting it from the ADF GUI. Change the Basis set, XC-Funtional, Numerical quality and Solvent if required. Upload the files using FileZilla to the Lisa server. Connect to the Lisa server in the terminal. A typical command on the server requires the following commands in the terminal.

module load pre2019

module load ADF

module load ADF/2019.102-intelmpi

sbatch input-filename.txt

Calculate each geometry in the terminal. Once the geometry optimisations are done, download the logfile and the output file using FileZilla. Extract the geometries from the final geometry cycle in the logfile and save as .mol2 files with an appropriate name. The bonding energy and the excited state energy are available at the end of the logfile. Other properties such as excited state dipole moment can be found in the output by searching for ''excited state dipole moment'' in the final geometry cycle.

SOC and bonding energy calculations

The SOC and bonding energy calculations make use of a standard template file for the calculations *SOC.txt.* Change the Basis set, XC-Funtional, Numerical quality and Solvent in this template input file if required. To do a dihedral scan of a particular state simply go to the ADF GUI and then go to ''Help ''. Select ''Command Line''. This opens the ADF command line terminal. In this terminal go to the folder with the .mol2 file, which is required for the scan. In the bash file *Amsprep-SOC-coordinates.txt*, specify the .mol2 file being used for the scan, the atom numbers between which the dihedral angle is defined and the range of the dihedral angle for the scan. Execute the bash file *Amsprep-SOC-coordinates.txt* in the command line by typing *sh Amsprep-SOC-coordinates.txt*. This bash file makes use of the amsprep environment in AMS to generate the coordinates for the range of dihedral angles of a particular state, and thus moves the coordinate files to a separate folder for each dihedral angle. Unfortunately, the

environment is limited to solvents. Using the Matlab 2018b file *replace_coordinates_SOC.m*, the generated coordinates replace the coordinates in the standard template file for SOC calculation. Repeat the procedure for other states and solvents in separate folders. Upload these folders with the input files on the Lisa server. Run the file *soc_energy_calculations.txt* by typing *sh soc_energy_calculations.txt* in the terminal connected to the Lisa server. This file contains the terminal commands for loading and submitting batch jobs for the SOC and energy calculations at each dihedral angle. Once the calculations are finished, download the output and logfile from the server. The excitation energy and spin orbit coupling can be found in the output file by going to "Response Properties" and then "Spin-orbit matrix". The data is analysed in the *SOC_PyrDMA.m* and *Energy_PyrDMA.m* files, which makes the required SOC and excitation energy plots.

Electronic coupling calculations

The calculation for the electronic coupling require fragments of the donor and acceptor in the locally excited state. The fragments are generated using the following procedure.

Fragments generation

The optimized excited state geometry of the precursor state (S_2) was saved as a .mol2 file within ADF. This file was imported into Chimera. Then the CH_2 group was selected (Ctrl-Shift) and then deleted together with its bonds. With the "add H" command in the "Structure Editing" menu, hydrogen atoms were added, which had the appropriate direction and length.

Main Electronic coupling calculations

The Electronic coupling calculations make use of three standard template files. *PyrDMA-Fragment-a.txt*, *PyrDMA-Fragment-b.txt* and *PyrDMA-vab.txt.* Change the Basis set, XC-Funtional, Numerical quality and Solvent in these template files if required. To do a dihedral scan of the electronic coupling, simply go to the ADF GUI and then "Help". Select "Command Line". Go to the folder with the .mol2 file of the PyrDMA in the S2 state, which is required for the scan. In the bash file *Amsprep-CT-coordinates.txt*, specify the .mol2 file being used for the scan, the range of atom number belonging to each fragment, the atom numbers between which the dihedral angle is defined and the range of the dihedral angle. Execute the bash file *Amsprep-CT-coordinates.txt* in the command line by typing *sh Amsprep-CT-coordinates.txt*. This bash file generates the coordinates for the range of dihedral angles of the locally excited state S_2 and moves the coordinate files to a separate folder for each dihedral

angle. Using the Matlab 2018b file *replace_coordinates_CT.m*, the generated coordinates replace the coordinates in the three standard template files for the electronic coupling calculation. Repeat the procedure for other solvents in separate folders. Upload these folders with the input files on the Lisa server using FileZilla. Run the file *CT_calculations-fragment-a.txt* and *CT_calculations-fragment-b.txt* in the terminal connected to the Lisa server. This file contains the terminal commands for loading and submitting batch jobs for the fragment calculations at each dihedral angle. Once the calculations are finished, run *CT_calculations-vab.txt*. These calculate the charge transfer integrals between fragment a (pyrene) and fragment b (dimethylaniline) for each dihedral angle. Download the final output files from the server. The charge transfer integrals can be found by searching for "V for hole transfer:". The data is analysed in the *CT_PyrDMA.m*, which makes the finale electronic coupling plots.

Internal reorganisation energy calculations

For the internal reorganisation energy calculations, we need to look at equation 5,6 and 7. First, we import the starting coordinates of PyrDMA into the ADF GUI. The donor and acceptor defined for the internal reorganisation energy are N,N-dimethylaniline for the donor and 1-methylpyrene for the acceptor. The starting coordinates for the donor and acceptor can be obtained by clicking the bond connecting these fragments and deleting this bond by pressing *Ctrl+x*. Afterwards, hydrogen atoms can be added by pressing *Ctrl+E.* Copy the coordinates for donor and acceptor from the ADF GUI and separately perform an optimisation on the donor and acceptor. The starting coordinates for the donor and acceptor are now obtained. Neutral and charged geometry optimisation need to be calculated for the donor and acceptor. This can be achieved by using the standard geometry optimisation input files. Replace the starting coordinates in the input files, set the calculation to Unrestricted and change the Charge key word to either Charge 0 0 for neutral geometry optimisations or Charge 0 $\pm$1 for charged geometry optimisation of the cation/anion. Place these input files in separate folders, upload on the Lisa server and run the calculations. When the calculations are finished, download the logfiles and extract the geometries from the final geometry cycle at the end of the logfile and save these as .mol2 files. To calculate the internal reorganisation energy, 4 single point calculations are required for both the donor and acceptor. The single point calculations can be done by using the standard template file *SP-energy.txt* and changing the coordinates to the charged/neutral geometry of the anion or cation. For each single point calculation change, the coordinates and the charge in the file according equation 5 and 6. Place each single point calculation in a separate folder and upload these files on the Lisa served using FileZilla. Use the 2019 version of ADF to run the single point calculations. Once the calculations are finished, download the logfiles containing the bonding energies from the server and extract these bonding energies. Use equation S2, S3 and S4 to calculate the reorganisation energy for the donor-

acceptor and the total internal reorganisation energy. Please take into account that the coordinates of the ''neutral'' state of the acceptor should be the coordinates of the locally excited state of pyrene S_1 for charge separation. For charge recombination to the ground state S_0, the coordinates of the ''neutral'' state of the acceptor are the coordinates of the ground state of the acceptor. For charge recombination to the triplet state T_1, the coordinates of the ''neutral'' state of the acceptor are the coordinates of the triplet state of the acceptor.

List of abbreviations

TD-DFT	Time-dependent density functional theory
ADF	Amsterdam Density Functional
DMA	dimethylaniline
Pyr	pyrene
NHX	n-hexane
ACN	acetonitrile
CS	charge separation
CR	charge recombination
ISC	Intersystem crossing
CT	Charge transfer
SO-ISC	Spin-orbit coupling intersystem crossing
SOCT-ISC	Spin-orbit charge transfer intersystem crossing
MO	Molecular orbital
SOC	Spin-orbit coupling
SOCME	Spin-orbit coupling matrix element
GS	Ground-state
LE	Local excitation
T_n	triplet (state)
S_n	Singlet (state)
^{n}CS	Charge separated (state)
V_{CT}	Electronic coupling
V_{SOC}	Spin orbit coupling
T	Temperature
$\hbar$	reduced Planck constant
k_B	Boltzmann constant
c	Speed of light
λ	Reorganization Energy
τ	lifetime of a state
PES	Potential Energy Surface
ΔG	Gibbs free energy
S	Huang Rhys factor
FCWD	Frank Condon Weighted Density
f_{SOC}	Oscillator strength
TDA	Tamm-Dancoff approximation
NTO	Natural Transition Orbital
COSMO	Conductor-like screening model
ZORA	Zero-order regular approximation

References

[1] Gao, X.; Bai, S.; Fazzi, D.; Niehaus, T.; Barbatti, M.; Thiel, W. Evaluation of Spin-Orbit Couplings with Linear-Response Time-Dependent Density Functional Methods. *J. Chem. Theory Comput.* **2017**, *13* (2), 515–524. https://doi.org/10.1021/acs.jctc.6b00915.

[2] Gibbons, D. J.; Farawar, A.; Mazzella, P.; Leroy-Lhez, S.; Williams, R. M. Making Triplets from Photo-Generated Charges: Observations, Mechanisms and Theory. *Photochem. Photobiol. Sci.* **2020**, *19* (2), 136–158. https://doi.org/10.1039/c9pp00399a.

[3] Schultz, D. M.; Yoon, T. P. Solar Synthesis: Prospects in Visible Light Photocatalysis. *Science (80).* **2014**, *343* (6174). https://doi.org/10.1126/science.1239176.

[4] Smith, M. B.; Michl, J. Singlet Fission. *Chem. Rev.* **2010**, *110* (11), 6891–6936. https://doi.org/10.1021/cr1002613.

[5] Celli, J. P.; Spring, B. Q.; Rizvi, I.; Evans, C. L.; Samkoe, K. S.; Verma, S.; Pogue, B. W.; Hasan, T. Imaging and Photodynamic Therapy: Mechanisms, Monitoring, and Optimization. *Chem. Rev.* **2010**, *110* (5), 2795–2838. https://doi.org/10.1021/cr900300p.

[6] Zhao, J.; Wu, W.; Sun, J.; Guo, S. Triplet Photosensitizers: From Molecular Design to Applications. *Chem. Soc. Rev.* **2013**, *42* (12), 5323–5351. https://doi.org/10.1039/c3cs35531d.

[7] Filatov, M. A. Heavy-Atom-Free BODIPY Photosensitizers with Intersystem Crossing Mediated by Intramolecular Photoinduced Electron Transfer. *Org. Biomol. Chem.* **2019**, *18* (1), 10–27. https://doi.org/10.1039/c9ob02170a.

[8] Lv, M.; Yu, Y.; Sandoval-Salinas, M. E.; Xu, J.; Lei, Z.; Casanova, D.; Yang, Y.; Chen, J. Engineering the Charge-Transfer State to Facilitate Spin–Orbit Charge Transfer Intersystem Crossing in Spirobis[Anthracene]Diones. *Angew. Chemie - Int. Ed.* **2020**, *59*, 2–8. https://doi.org/10.1002/anie.202009439.

[9] El-Sayed, M. A. Spin-Orbit Coupling and the Radiationless Processes in Nitrogen Heterocyclics. *J. Chem. Phys* **1963**, *38*, 2834. https://doi.org/10.1063/1.1733610.

[10] Okada, T.; Karaki, I.; Matsuzawa, E.; Mataga, N.; Sakata, Y.; Misumi, S. Ultrafast Intersystem Crossing in Some Intramolecular Heteroexcimers. *J. Phys. Chem.* **1981**, *85* (26), 3957–3960. https://doi.org/10.1021/j150626a002.

[11] Van Willigen, H; Guilford Jones, I.; Farahat, M. S. Time-Resolved EPR Study of Photoexcited Triplet-State Formation in Electron-Donor-Substituted Acridinium Ions. *J. Phys. Chem.* **1996**, *100* (9), 3312–3316. https://doi.org/10.1021/JP953176+.

[12] Dance, Z. E. X.; Mickley, S. M.; Wilson, T. M.; Ricks, A. B.; Scott, A. M.; Ratner, M. A.; Wasielewski, M. R. Intersystem Crossing Mediated by Photoinduced Intramolecular Charge Transfer: Julolidine - Anthracene Molecules with Perpendicular π Systems. *J. Phys. Chem. A.* **2008**, *112* (18), 4194–4201. https://doi.org/10.1021/jp800561g.

[13] Buck, J. T.; Boudreau, A. M.; DeCarmine, A.; Wilson, R. W.; Hampsey, J.; Mani, T. Spin-Allowed Transitions Control the Formation of Triplet Excited States in Orthogonal Donor-Acceptor Dyads. *Chem.* **2019**, *5* (1), 138–155. https://doi.org/10.1016/j.chempr.2018.10.001.

[14] Sasikumar, D.; John, A. T.; Sunny, J.; Hariharan, M. Access to the Triplet Excited States of Organic Chromophores. *Chem. Soc. Rev.* **2020**, *49* (17), 6122–6140. https://doi.org/10.1039/d0cs00484g.

[15] Nguyen, V.-N.; Yan, Y.; Zhao, J.; Yoon, J. Heavy-Atom-Free Photosensitizers: From Molecular Design to Applications in the Photodynamic Therapy of Cancer. *Acc. Chem. Res.* **2021**, *54* (1), 207–220. https://doi.org/10.1021/acs.accounts.0c00606.

[16] Lowry, T. H.; Richardson, K. S.; *Mechanism and theory in organic chemistry*. New York : Harper & Row, 1987

[17] Williams, R. M.; Zwier, J. M.; Verhoeven, J. W. Photoinduced Intramolecular Electron Transfer in a Bridged C60 (Acceptor)-Aniline (Donor) System; Photophysical Properties of the First “Active” Fullerene Diad. *J. Am. Chem. Soc.* **1995**, *117* (14), 4093–4099. https://doi.org/10.1021/ja00119a025.

[18] Williams, R. M.; Koeberg, M.; Lawson, J. M.; An, Y.-Z.; Rubin, Y.; Paddon-Row, M. N.; Verhoeven, J. W. Photoinduced Electron Transfer to $C_{60}C_{60}$ across Extended 3- and 11-Bond Hydrocarbon Bridges: Creation of a Long-Lived Charge-Separated State. *J. Org. Chem.* **1996**, *61* (15), 5055–5062. https://doi.org/10.1021/jo960678q.

[19] Williams, R. M.; Verhoeven, J. W. Fluorescence of Fullerene-C70 and Its Quenching by Long-Range Intermolecular Electron Transfer. *Chem. Phys. Lett.* **1992**, *194* (4), 6.

[20] Cardeynaels, T.; Paredis, S.; Deckers, J.; Brebels, S.; Vanderzande, D.; Maes, W.; Champagne, B. Finding the Optimal Exchange–Correlation Functional to Describe the Excited State Properties of Push–Pull Organic Dyes

Designed for Thermally Activated Delayed Fluorescence. *Phys. Chem. Chem. Phys.* **2020**, *22* (28), 16387–16399. https://doi.org/10.1039/D0CP02409K.
[21] Kelber, J. B.; Panjwani, N. A.; Wu, D.; Gómez-Bombarelli, R.; Lovett, B. W.; Morton, J. J. L.; Anderson, H. L. Synthesis and Investigation of Donor–Porphyrin–Acceptor Triads with Long-Lived Photo-Induced Charge-Separate States. *Chem. Sci.* **2015**, *6* (11), 6468–6481. https://doi.org/10.1039/C5SC01830G.
[22] Chaudhuri, S.; Hedström, S.; Méndez-Hernández, D. D.; Hendrickson, H. P.; Jung, K. A.; Ho, J.; Batista, V. S. Electron Transfer Assisted by Vibronic Coupling from Multiple Modes. *J. Chem. Theory Comput.* **2017**, *13* (12), 6000–6009. https://doi.org/10.1021/acs.jctc.7b00513.
[23] Plasser, F.; Lischka, H. Analysis of Excitonic and Charge Transfer Interactions from Quantum Chemical Calculations. *J. Chem. Theory Comput.* **2012**. https://doi.org/10.1021/ct300307c.
[24] Scherer, P. O. J. Intramolecular Reorganization of the Electron Donor *N* , *N* -Dimethylaniline. [†]. *J. Phys. Chem. A* **2003**, *107* (40), 8327–8329. https://doi.org/10.1021/jp027855d.
[25] Scherer, P. O. J.; Tachiya, M. Computer Simulation Studies of Electron Transfer Parameters for Cyanoanthracene/N,N-Dimethylaniline Solutions. *J. Chem. Phys.* **2003**, *118* (9), 4149–4156. https://doi.org/10.1063/1.1541617.
[26] Schomburg, H.; Staerk, H.; Weller, A. Electron Transfer Reactions And Inhibition Of Triplet State Formation In Mlxed Fluorescence Quencher Experlments. Chem. Phys. Lett.., 22 (1973), p. 1
[27] Vân Anh, N.; Schlosser, F.; Groeneveld, M. M.; van Stokkum, I. H. M.; Würthner, F.; Williams, R. M. Photoinduced Interactions in a Pyrene-Calix[4]Arene-Perylene Bisimide Dye System: Probing Ground-State Conformations with Excited-State Dynamics of Charge Separation and Recombination. *J. Phys. Chem. C*. **2009**, *113* (42), 18358–18368. https://doi.org/10.1021/jp9055279.
[28] Leroy-lhez, S.; Belin, C.; D'aleo, A.; Williams, R. M.; De Cola, L.; Fages, F. Extending Excited-State Lifetimes by Interchromophoric Triplet-State Equilibration in a Pyrene-Ru(II)Diimine Dyad System. *Supramol. Chem.* **2003**, *15* (7–8), 627–637. https://doi.org/10.1080/10610270310001605214
[29] Montalti, M.; Credi, A.; Prodi, L.; Gandolfi, M. T. *Handbook of Photochemistry*; CRC Press, 2006.
[30] Rujkorakarn, R.; Tanaka, F. Three-Dimensional Representations of Photo-Induced Electron Transfer Rates in Pyrene-(CH2)n-N,N'-Dimethylaniline Systems Obtained by Three Electron Transfer Theories. *J. Mol. Graph. Model.* **2009**, *27* (5), 571–577. https://doi.org/10.1016/j.jmgm.2008.09.008.
[31] Zhu, Q.; Feng, S.; Guo, X.; Chen, X.; Zhang, J. Strategy for Tuning the Up-Conversion Intersystem Crossing Rates in a Series of Organic Light-Emitting Diodes Emitters Relevant for Thermally Activated Delayed Fluorescence. *Spectrochim. Acta - Part A Mol. Biomol. Spectrosc.* **2019**, *221*, 117214. https://doi.org/10.1016/j.saa.2019.117214.
[32] SCM. Amsterdam Modeling Suite Making Computational Chemistry Work For You Software for Chemistry & Materials https://www.scm.com/ (accessed Feb 9, **2021**).
[33] Hou, Y.; Biskup, T.; Rein, S.; Wang, Z.; Bussotti, L.; Russo, N.; Foggi, P.; Zhao, J.; Di Donato, M.; Mazzone, G.; et al. Spin-Orbit Charge Recombination Intersystem Crossing in Phenothiazine-Anthracene Compact Dyads: Effect of Molecular Conformation on Electronic Coupling, Electronic Transitions, and Electron Spin Polarizations of the Triplet States. *J. Phys. Chem. C.* **2018**, *122* (49), 27850–27865. https://doi.org/10.1021/acs.jpcc.8b08965.
[34] Hou, Y.; Zhang, X.; Chen, K.; Liu, D.; Wang, Z.; Liu, Q.; Zhao, J.; Barbon, A. Charge Separation, Charge Recombination, Long-Lived Charge Transfer State Formation and Intersystem Crossing in Organic Electron Donor/Acceptor Dyads. *J. Mater. Chem. C.* **2019**, *7* (39), 12048–12074. https://doi.org/10.1039/c9tc04285g.
[35] Wen, S. H.; Li, A.; Song, J.; Deng, W. Q.; Han, K. L.; Goddard, W. A. First-Principles Investigation of Anistropic Hole Mobilities in Organic Semiconductors. *J. Phys. Chem. B* **2009**, *113* (26), 8813–8819. https://doi.org/10.1021/jp900512s.
[36] Üngördü, A. Electronic, Optical, and Charge Transfer Properties of Porphyrin and Metallated Porphyrins in Different Media. *Int. J. Quantum Chem.* **2020**, *120* (6). https://doi.org/10.1002/qua.26128.
[37] Monti, A.; De Groot, H. J. M.; Buda, F. In-Silico Design of a Donor-Antenna-Acceptor Supramolecular Complex for Photoinduced Charge Separation. *J. Phys. Chem. C.* **2014**, *118* (29), 15600–15609. https://doi.org/10.1021/jp505105a.
[38] Martínez, J. P.; Trujillo-González, D. E.; Götz, A. W.; Castillo-Alvarado, F. L.; Rodríguez, J. I. Effects of Dispersion Forces on Structure and Photoinduced Charge Separation in Organic Photovoltaics. *J. Phys. Chem. C.* **2017**, *121* (37), 20134–20140. https://doi.org/10.1021/acs.jpcc.7b05107.
[39] Marcus, R. A. On the Theory of Electron-Transfer Reactions. VI. Unified Treatment for Homogeneous and Electrode Reactions. *J. Chem. Phys.* **1965**, *43* (2), 679–701. https://doi.org/10.1063/1.1696792.

[40] Nelsen, S. F.; Blackstock, S. C.; Kim, Y. Estimation of Inner Shell Marcus Terms for Amino Nitrogen Compounds by Molecular Orbital Calculations. *J. Am. Chem. Soc.* **1987**, *109* (3), 677–682. https://doi.org/10.1021/ja00237a007.
[41] Peach, M. J. G.; Benfield, P.; Helgaker, T.; Tozer, D. J. Excitation Energies in Density Functional Theory: An Evaluation and a Diagnostic Test. *J. Chem. Phys.* **2008**, *128* (4), 044118. https://doi.org/10.1063/1.2831900.
[42] Marcus, R. A. Electron Transfer Reactions in Chemistry: Theory and Experiment (Nobel Lecture). *Angew. Chem. Int. Ed. Engl.* **1993**, *32* (8), 1111–1121. https://doi.org/10.1002/anie.199311113.
[43] Verhoeven, J. W.; Van Ramesdonk, H. J.; Groeneveld, M. M.; Benniston, A. C.; Harriman, A. Long-Lived Charge-Transfer States in Compact Donor-Acceptor Dyads. *ChemPhysChem*. November 11, **2005**, pp 2251–2260. https://doi.org/10.1002/cphc.200500029.
[44] Hu, Y.; Hou, Y.; Wang, Z.; Li, Y.; Zhao, J. 3,5-Anthryl-Bodipy Dyad/Triad: Preparation, Effect of F-B-F Induced Conformation Restriction on the Photophysical Properties, and Application in Triplet-Triplet-Annihilation Upconversion. *J. Chem. Phys.* **2020**, *153* (22), 224304. https://doi.org/10.1063/5.0025224.
[45] D'Souza, F.; Subbaiyan, N. K.; Xie, Y.; Hill, J. P.; Ariga, K.; Ohkubo, K.; Fukuzumi, S. Anion-Complexation-Induced Stabilization of Charge Separation. *J. Am. Chem. Soc.* **2009**, *131* (44), 16138–16146. https://doi.org/10.1021/ja9048306.
[46] Marian, C. M. Spin-Orbit Coupling and Intersystem Crossing in Molecules. *Wiley Interdiscip. Rev. Comput. Mol. Sci.* **2012**, *2* (2), 187–203. https://doi.org/10.1002/wcms.83.
[47] Beljonne, D.; Shuai, Z.; Pourtois, G.; Bredas, J. L. Spin-Orbit Coupling and Intersystem Crossing in Conjugated Polymers: A Configuration Interaction Description. *J. Phys. Chem. A.* **2001**, *105* (15), 3899–3907. https://doi.org/10.1021/jp010187w.
[48] Yang, B.; Huang, S.; Luo, S. A. Theoretical Research on Intersystem Crossing, Radiative and Nonradiative Rates of Cyclometalated Platinum(II) Complexes. *Theor. Chem. Acc.* **2019**, *138* (6), 77. https://doi.org/10.1007/s00214-019-2466-6.
[49] Samanta, P. K.; Kim, D.; Coropceanu, V.; Brédas, J. L. Up-Conversion Intersystem Crossing Rates in Organic Emitters for Thermally Activated Delayed Fluorescence: Impact of the Nature of Singlet vs Triplet Excited States. *J. Am. Chem. Soc.* **2017**, *139* (11), 4042–4051. https://doi.org/10.1021/jacs.6b12124.
[50] Ruhoff, P. T.; Ratner, M. A. Algorithms for Computing Franck-Condon Overlap Integrals. *Int. J. Quantum Chem.* **2000**, *77* (1), 383–392. https://doi.org/10.1002/(SICI)1097-461X(2000)77:1<383::AID-QUA38>3.0.CO;2-0.
[51] Martin, R. L. Natural Transition Orbitals. *J. Chem. Phys.* **2003**, *118*, 4775. https://doi.org/10.1063/1.1558471.
[52] Liu, T.; Troisi, A. Absolute Rate of Charge Separation and Recombination in a Molecular Model of the P3HT/PCBM Interface. *J. Phys. Chem. C.* **2011**, *115* (5), 2406–2415. https://doi.org/10.1021/jp109130y.
[53] Daswani, U.; Singh, U.; Sharma, P.; Kumar, A. From Molecules to Devices: A DFT/TD-DFT Study of Dipole Moment and Internal Reorganization Energies in Optoelectronically Active Aryl Azo Chromophores. *J. Phys. Chem. C.* **2018**, *122* (26), 14390–14401. https://doi.org/10.1021/acs.jpcc.8b04070.
[54] Turro, N. J.; Ramamurthy, V.; Scaiano, J. C. *Principles of Molecular Photochemistry: An Introduction*; University Science Books, 2009.
[55] Ronca, E.; De Angelis, F.; Fantacci, S. Time-Dependent Density Functional Theory Modeling of Spin-Orbit Coupling in Ruthenium and Osmium Solar Cell Sensitizers. *J. Phys. Chem. C.* **2014**, *118* (30), 17067–17078. https://doi.org/10.1021/jp500869r.
[56] Wang, Z.; Sukhanov, A. A.; Toffoletti, A.; Sadiq, F.; Zhao, J.; Barbon, A.; Voronkova, V. K.; Dick, B. Insights into the Efficient Intersystem Crossing of Bodipy-Anthracene Compact Dyads with Steady-State and Time-Resolved Optical/Magnetic Spectroscopies and Observation of the Delayed Fluorescence. *J. Phys. Chem. C.* **2019**, *123* (1), 265–274. https://doi.org/10.1021/acs.jpcc.8b10835.
[57] Letrun, R.; Lang, B.; Yushchenko, O.; Wilcken, R.; Svechkarev, D.; Kolodieznyi, D.; Riedle, E.; Vauthey, E. Excited-State Dynamics of a Molecular Dyad with Two Orthogonally-Oriented Fluorophores. *Phys. Chem. Chem. Phys.* **2018**, *20* (48), 30219–30230. https://doi.org/10.1039/c8cp05356a.
[58] Gonzalez-Vazquez, J. P.; Burn, P. L.; Powell, B. J. Interplay of Zero-Field Splitting and Excited State Geometry Relaxation in Fac-Ir(Ppy)3. *Inorg. Chem.* **2015**, *54* (21), 10457–10461. https://doi.org/10.1021/acs.inorgchem.5b01918.
[59] Minaev, B.; Ågren, H. Theoretical DFT Study of Phosphorescence from Porphyrins. *Chem. Phys.* **2005**, *315* (3), 215–239. https://doi.org/10.1016/j.chemphys.2005.04.017.
[60] Tsvirko, M., Solovjev, K., Gradyushko, A., Dvornikov, S. Phosphorescence of Porphyrin Free Bases and Their Complexes with Light Metals. *Opt. Spectrosc.* **1975**, *38*, 400.

[61] Pye, C. C.; Ziegler, T. An Implementation of the Conductor-like Screening Model of Solvation within the Amsterdam Density Functional Package. *Theor. Chem. Acc.* **1999**, *101* (6), 396–408. https://doi.org/10.1007/s002140050457.
[62] Sutton, C.; Sears, J. S.; Coropceanu, V.; Brédas, J. L. Understanding the Density Functional Dependence of DFT-Calculated Electronic Couplings in Organic Semiconductors. *J. Phys. Chem. Lett.* **2013**, *4* (6), 919–924. https://doi.org/10.1021/jz3021292.
[63] Dreuw, A.; Head-Gordon, M. Failure of Time-Dependent Density Functional Theory for Long-Range Charge-Transfer Excited States: The Zinc bacteriochlorin-Bacteriochlorin and Bacteriochlorophyll-Spheroidene Complexes. *J. Am. Chem. Soc.* **2004**, *126* (12), 4007–4016. https://doi.org/10.1021/ja039556n.
[64] Sun, H.; Hu, Z.; Zhong, C.; Chen, X.; Sun, Z.; Brédas, J. L. Impact of Dielectric Constant on the Singlet-Triplet Gap in Thermally Activated Delayed Fluorescence Materials. *J. Phys. Chem. Lett.* **2017**, *8* (11), 2393–2398. https://doi.org/10.1021/acs.jpclett.7b00688.
[65] Chen, K.; Zhao, J.; Li, X.; Gurzadyan, G. G. Anthracene-Naphthalenediimide Compact Electron Donor/Acceptor Dyads: Electronic Coupling, Electron Transfer, and Intersystem Crossing. *J. Phys. Chem. A.* **2019**, *123* (13), 2503–2516. https://doi.org/10.1021/acs.jpca.8b11828.
[66] Isukapalli, S. V. K.; Lekshmi, R. S.; Samanta, P. K.; Vennapusa, S. R. Formation of Excited Triplet States in Naphthalene Diimide and Perylene Diimide Derivatives: A Detailed Theoretical Study. *J. Chem. Phys.* **2020**, *153* (12), 124301. https://doi.org/10.1063/5.0012476.
[67] Imran, M.; Sukhanov, A. A.; Wang, Z.; Karatay, A.; Zhao, J.; Mahmood, Z.; Elmali, A.; Voronkova, V. K.; Hayvali, M.; Xing, Y. H.; et al. Electronic Coupling and Spin-Orbit Charge-Transfer Intersystem Crossing in Phenothiazine-Perylene Compact Electron Donor/Acceptor Dyads. *J. Phys. Chem. C.* **2019**, *123* (12), 7010–7024. https://doi.org/10.1021/acs.jpcc.8b12040.
